# Supplementary material for: Identification of photoperiod-induced specific miRNAs in the adrenal glands of Sunite sheep (Ovis aries)
Source: Front Vet Sci. 2022 Jul 22;9:888207. doi: 10.3389/fvets.2022.888207 (PMC9354845; doi:10.3389/fvets.2022.888207)
Supplement: Supplementary file 1 [file Data_Sheet_1.ZIP › Supplementary file/miRDeep2 file.zip!/miRDeep2 file/Novel_Structure/LP42A3/Novel_379.pdf]

Provisional ID	:	NC_019461.2_6976
Score total	:	0.9
Score for star read(s)	:	-1.3
Score for read counts	:	0
Score for mfe	:	0.6
Score for randfold	:	1.6
Score for cons. seed	:	
Total read count	:	23
Mature read count	:	22
Loop read count	:	0
Star read count	:	1

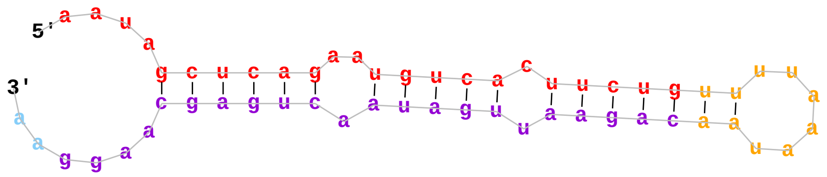

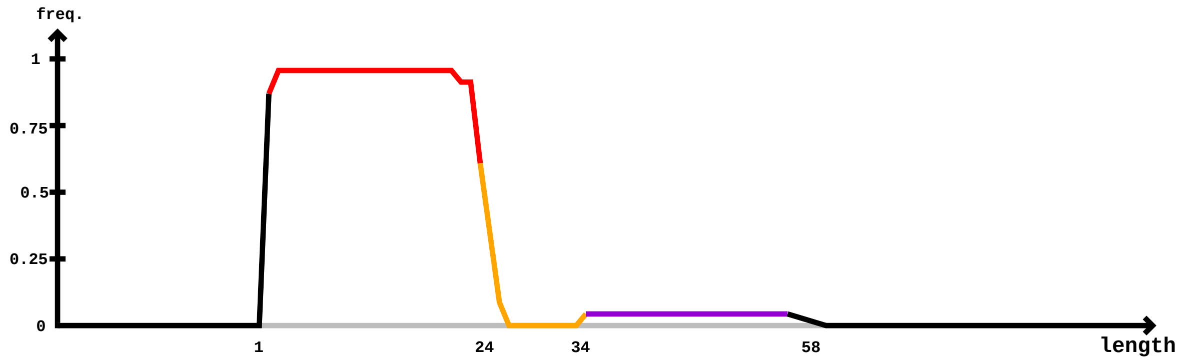

Mature **Star**

```
5'- uuuguaauucuuuacuguggaauagcucagaaugucacuucuguuuuaaauaacagaauugauaacugagcaaggaaacguaauuuggauuauaaauucuugcuuuaauaaa   -3'    obs
    uuuguaauucuuuacuguggaauagcucagaaugucacuucuguuuuaaauaacagaauugauaacugagcaaggaaacguaauuuggauuauaaauucuugcuuuaauaaa          exp
    ((((((((((.((((.((......((((((..(((((.(((((((......))))))).))))).))))))......))))))...))))))))))................   reads    mm    sample
    ....................aauagcucagaaugucacuu........................................................................   1        0     seq
    ....................aauagcucagaaugucacuucu......................................................................   7        0     seq
    ....................aauagcucagaaugucacuucug.....................................................................   12       0     seq
    .....................auagcucagaaugucacuucuguu...................................................................   2        0     seq
    .....................................................cagaauugauaacugagcaagg.....................................   1        0     seq
```
